# Phenolic Metabolites Protocatechuic Acid and Vanillic Acid Improve Nitric Oxide Bioavailability via the Akt-eNOS Pathway in Response to TNF-α Induced Oxidative Stress and Inflammation in Endothelial Cells

**DOI:** 10.3390/metabo14110613

**Published:** 2024-11-11

**Authors:** Joseph Festa, Aamir Hussain, Zakia Al-Hareth, Stephen J. Bailey, Harprit Singh, Mariasole Da Boit

**Affiliations:** 1 Leicester School of Allied Health Sciences, De Montfort University, The Gateway, Leicester LE1 9BH, UK; p2551377@alumni365.dmu.ac.uk (J.F.); aamir@researchtribe.org (A.H.); harprit.singh@dmu.ac.uk (H.S.); 2 The Jenner Institute, University of Oxford, ORCRB, Headington, Oxford OX3 7DQ, UK; zakia.alhareth@ndm.ox.ac.uk; 3 School of Sport, Exercise and Health Sciences, Loughborough University, Loughborough LE11 3TU, UK; s.bailey2@lboro.ac.uk

**Keywords:** Cyanidin-3-glucoside, endothelial nitric oxide synthase, nitric oxide, protocatechuic acid, vanillic acid

## Abstract

**Background/Objectives**: Reduced nitric oxide (NO) bioavailability secondary to excess-superoxide-driven oxidative stress is central to endothelial dysfunction. Previous studies suggest that phenolic metabolites may improve NO bioavailability, yet limited research is available in response to an inflammatory mediator. Therefore, we assessed the effects of cyanidin-3-glucoside (C3G) and its phenolic metabolites protocatechuic acid (PCA) and vanillic acid (VA) on NO bioavailability in a TNF-α induced inflammatory environment. **Methods**: Primary human umbilical vein endothelial cells (HUVECs) were supplemented with either C3G, PCA, or VA at 1 μM for 24 h before being stimulated with TNF-α 20 ng/mL for an additional 24 h. Measurements included cell viability, apoptosis, reactive oxygen species (ROS), nitrite concentrations, and endothelial nitric oxide synthase (eNOS) and Akt at the mRNA and protein level. **Results**: Phenolic metabolites did not increase the eNOS expression or nitrite levels in the unstimulated environment; rather, the metabolites mediated NO bioavailability in response to TNF-α induced oxidative stress, with increased viability, eNOS mRNA, phosphorylation, and nitrite levels. **Conclusions**: Phenolic metabolites, in the presence of TNF-α, can improve NO bioavailability at physiologically relevant concentrations via the Akt-eNOS pathway. This demonstrates that the induction of inflammation is a prerequisite for phenolic metabolites to promote protective properties in endothelial cells by activating the Akt-eNOS pathway.

## 1. Introduction

Nitric oxide (NO) is a multi-functional signaling molecule that plays a vital role in maintaining adhesion molecule expression, vascular tone, platelet aggregation, smooth muscle cell proliferation, and vascular homeostasis [1]. The generation of NO in the vascular endothelium occurs via the upregulation of the Akt–endothelial nitric oxide synthase (eNOS) enzyme pathway within endothelial cells [2]. Reduced NO bioactivity is linked to endothelial dysfunction (ED), an important process underlying the development and progression of atherosclerosis, as well as many cardiovascular diseases (CVD) [3]. In part, reduced NO synthesis and bioavailability with ED may be linked to elevated reactive oxygen species (ROS) production, oxidative stress and inflammation [4]. Indeed, oxidative stress and inflammation have been linked to increased arginase activity and thereby lower L-arginine substrate levels for eNOS; the increased formation of the L-arginine analog and competitive eNOS inhibitor asymmetric dimethylarginine; and the oxidation of the eNOS co-factor tetrahydrobiopterin, can lead to eNOS uncoupling, whereby the five-electron oxidation of L-arginine is incomplete, with the ROS superoxide formed instead of NO [5]. Moreover, since NO is a free radical, due to its unpaired electron, NO can react with superoxide to scavenge NO, leading to lower NO bioavailability and the formation of peroxynitrite, which is a highly reactive molecule that can result in nitrative stress [4]. Interventions to lower oxidative stress, therefore, have the potential to improve NO synthesis, bioavailability, and signaling. One simple dietary approach is to increase the consumption of fruits and vegetables, which is associated with a decrease in the risk of CVD, due, at least in part, to their high content of polyphenols, particularly anthocyanins, which confer antioxidant effects [6,7,8]. Moreover, studies have shown improvements in different aspects of vascular function, such as endothelial function, the control of blood lipids, and platelet aggregation following the consumption of dietary flavonoids [9].

Cyanidin-3-glucoside (C3G) is a potent anthocyanin found in a variety of food products, including berries and berry extracts, which has displayed protective effects against stressors in endothelial cells [10]. Recent studies have suggested that C3G potentially exerts its functions on the body primarily through its metabolites [11,12], with more than 20 types of metabolites identified in human serum and urine after the consumption of C3G [13]. The highest mean concentration of a combination of metabolites detected in plasma, following a 500 mg bolus of ^13^C-labeled anthocyanins, was observed at 24 h post-consumption and totaled 4.38 μM [14]. Although the function and mechanism of C3G metabolites are not fully established, protocatechuic acid (PCA), vanillic acid (VA), and their derivates are emerging as some of the most bioactive metabolites of C3G due to their antioxidant and anti-inflammatory properties [15].

PCA is derived from the A and B rings of the original anthocyanin compound, wherein VA is the methylated form of PCA [16]. Despite this interesting emerging evidence to support the antioxidant activity of PCA and VA, little attention has been paid to their effects on endothelial cells [16]. A study by Edwards and colleagues found that C3G increased eNOS protein levels in human umbilical vein endothelial cells (HUVECs), wherein its phenolic metabolites, PCA and VA, did not increase the production of either the eNOS protein or NO [17]. In contrast, VA elicited reductions in superoxide production, which could subsequently decrease the scavenging of NO, suggesting a possible increase in NO bioavailability, rather than increasing the production of NO [17]. In addition, it has been shown that PCA may modulate the phosphorylation of eNOS and not its expression [18].

Adding to the limited in vitro data, animal studies have shown that VA can increase the plasma levels of NOx (nitrate and nitrite) in NO-deficient rats, by restoring the eNOS levels [19]. Furthermore, PCA has been shown to promote endothelial-dependent vasodilation by increasing the eNOS activity in ApoE−/− mice with established atherosclerosis but not in wild-type C57BL/6J mice not affected by atherosclerosis [20]. This might suggest that PCA and VA in isolation could restore eNOS expression as well as NO bioavailability, under pathological rather than normal conditions [21]. Thus, based on previous studies in mice, the induction of inflammation, or an established model of atherosclerosis, is a prerequisite for phenolic metabolites to promote endothelium-dependent protective properties. Nonetheless, in vitro findings elucidating the mechanisms of action of these phenolic metabolites, especially at physiologically relevant concentrations, in response to inflammation are limited. Therefore, the aim of this study was to assess the effects of a physiological concentration of 1 μM of C3G and its phenolic metabolites PCA and VA on the Akt-eNOS pathway and NO modulation following TNF-α induced inflammation.

## 2. Materials and Methods

### 2.1. Standards and Reagents

Cyanidin-3-glucoside (cat. number: 7084-24,4), PCA (cat. number: 99-50-3), and VA (cat. number: 121-34-6) were purchased from Sigma-Aldrich (Poole, UK). Standard solutions were prepared in 100% dimethyl sulfoxide (DMSO) (cat. number: 471267), which was also obtained from Sigma-Aldrich (molecular biology grade); the stock solution was prepared at a concentration of 100 mM and was diluted to working concentrations with a cell culture medium. Fetal calf serum (FCS) (cat. number: C8056) and H_2_O_2_ (cat. number: 216763) were also purchased from Sigma-Aldrich. Primary HUVECs (cat. number: C0035C), human large vessel endothelial cell basal media (cat. number: M200500), low serum growth supplement (LSGS) (cat. number: S00310), penicillin/streptomycin (cat. number: 15140122), annexin V–FITC (cat. number: A13199), 2′,7′-dichlorofluorescin diacetate (DCFHDA) (cat. number: D399), and phosphate-buffered saline (PBS) (cat. number: 10010023) were all purchased from ThermoFisher Scientific, Loughborough, UK.

### 2.2. Cell Culture and Stimulation

Primary HUVECs were maintained in human large vessel endothelial cell basal media supplemented with 2% LSGS, 10% heat-inactivated (65, 30 min) FCS, and 1% penicillin/streptomycin. Cells were cultured at ~1 × 10^6^ cells/mL in a T75 flask and incubated at 37 °C and 5% CO_2_. Every 48 h, cells were sub-cultured to maintain the intensity and were used at passage three for experiments. Prior to stimulation, cells were serum-starved for 1 h. HUVECs were stimulated with either C3G, PCA, or VA at 1 μM for 24 h +/− TNF-α at 20 ng/mL for an additional 24 h (48 h total). Negative and positive controls were diluted solutions of DMSO at 1:100 dilution and H_2_O_2_ at 1:1000, respectively.

### 2.3. Cell Viability, Apoptosis, and Intracellular ROS Assay

HUVECs were seeded in a 12-well cell culture plate at ~0.2 × 10^6^ cells/mL, maintained and stimulated as described above. After stimulation, adherent cells were trypsinized with 0.5% trypsin–EDTA for approximately 2 min, washed twice with cold PBS, and then centrifuged for 5 min at 600× *g*. For cell viability and apoptosis quantitation, cells were resuspended at ~1 × 10^6^ cells/mL in 400 μL 1X binding buffer with 5 μL of annexin V–FITC and were incubated in the dark for 15 min at 4 °C; then, propidium iodide was added for 5 min. Finally, cells were washed with 1 mL PBS, centrifuged at 600× *g* for 5 min, and resuspended in 1X binding buffer and were ready to be analyzed by flow cytometry. For ROS stimulation, cells were resuspended in 1 mL of PBS and 5 μM of DCFHDA was added to the cell suspension, followed by incubation at 37 °C for 30 min; finally, cells were fixed with 2% FBS before being flowed through the flow cytometry device. For both analyses, samples were analyzed using the BD Accuri C6 flow cytometer (Accuri, Ann Arbor, MI, USA).

### 2.4. Gene Expression

A two-step reverse transcriptase quantitative poly chain reaction (RT-qPCR) was conducted to detect the expression of genes of interest (GOIs). mRNA extraction, DNase treatment, cDNA synthesis, and RT-qPCR were performed using the Monarch^®^ Total RNA miniprep kit (cat. number: T2010S), Lunascript^®^ RT SuperMix kit (cat. number: E3010S), and Luna^®^ Universal qPCR Master Mix (cat. number: M3003S), all purchased from New England Biolabs, following the manufacturer’s instructions. The RNA quantity and purity were determined using a Nanodrop 1000^TM^ spectrophotometer with an A260 nm/A280 nm > 1.8 (Nanodrop, Thermofisher Scientific, Loughborough, UK). Extracted RNA was either used immediately or kept at −80 °C until further use.

Synthesized cDNA was either used for gene expression measurement or was kept at −20 °C for later use. The RT-qPCR cycling parameters were set as follows: 40 cycles of 94 °C denaturation (15 s), 60 °C annealing (30 s), and extension at 72 °C (60 s). A final dissociation stage was run to generate a melting curve for the verification of the amplification specificity. Controls included no template (NTC) and no reverse transcriptase (NRTC) controls and were conducted in tandem with all reactions. Gene expression was analyzed using SYBR Green Luna Universal qPCR master mix on the QuantStudio^TM^ 3 real-time PCR system (ThermoFisher Scientific, UK). The fold change in gene expression was analyzed using the ΔΔCq method, with GAPDH as the reference gene [22].

The efficiencies of primer pairs (eNOS forward 5′-GCT TCC CTT CCC TCT GTA AAT C-′3, reverse GGG CTG AAA CCA CCA TTA CT-′3, Akt forward 5′-GGA AAC TGA GGC TGG AGA TAA A, reverse CTC CCA AAG TGC CGA GAT TAT-′3, GAPDH forward 5′-TGA GAA CGG GAA GTC TGT CA, and reverse TCT CCA TGG TGG TGA AGA CG-′3) were evaluated by constructing a standard curve. Primers were designed using the NCBI primer design tool.

### 2.5. Immunoblot

The immunoblot protocol was carried out as previously described [23]. Briefly, stimulated cells were lysed in RIPA buffer to harvest the expressed proteins, and the resulting lysate was centrifuged at 13,000× *g* at 4 °C for 5 min. Proteins were denatured by the addition of Laemmli sample buffer containing β-mercaptoethanol (100 mM) and heated at 95 °C for 5 min before being resolved by 8% sodium dodecyl sulfate (SDS) page. Separated proteins were then transferred to a nitrocellulose membrane for antibody probing. Membranes were probed for p-Akt (Ser473) (cat. number: AF887), p-eNOS (ser1177) (cat. number: MAB9028), total Akt (cat. number: 281046), and eNOS (cat. number: AF950) (all purchased from R&D systems) at a 1:1000 dilution overnight at 4 °C. Following a wash step, the membranes were probed with secondary antibodies, either anti-rabbit (cat. number: HAF008), anti-goat (HAF017), or anti-mouse (HAF007), at a dilution of 1:2000. Incubation was performed at room temperature for 1 h, followed by an additional wash.

Bands were then visualized using chemiluminescence. The optical densities of the bands on the immunoblots were quantified using ImageStudioLite, and bands were normalized by using β-actin (cat. number: PA1-46296, ThermoFisher Scientific, UK) as a loading control. All data were expressed as a fold change with regard to either the negative control or TNF-α, as stated.

### 2.6. Measurement of Nitric Oxide Using Nitrite Assay

The short half-life (3–6 s) of NO makes it difficult to measure directly. Nitric oxide is oxidized to nitrite, as a more stable derivative, such that nitrite measurement is a surrogate for eNOS activity [24]. The supernatants of three independent experiments following the cells’ stimulation were harvested and centrifuged at 1500× *g* for 10 min to eliminate the cellular debris and were either immediately analyzed or kept at −80 °C for subsequent analysis. A Sievers NOA 280i (Analytix Ltd., Newcastle, UK) chemiluminescence-based NO analyzer was used to measure the nitrite in the collected cell culture supernatants. A 50 μL undiluted sample was injected into a purge vessel containing 5 mL of 4% (*w*/*v*) glacial acetic acid and sodium iodide solution, which reduced nitrite to NO gas. The resultant NO gas was carried into the reaction cells of the analyzer with inert nitrogen gas, where the NO gas reacted with ozone to form nitrogen dioxide in the excited state. Upon return to its ground state, the electronically excited nitrogen dioxide emitted a photon that was detected by a red-sensitive photomultiplier tube. The concentration of nitrite in the samples was calculated using a standard plot of known sodium nitrite concentrations.

### 2.7. Data Analysis

Statistical analysis was performed using GraphPad Prism version 9 (USA). The Shapiro–Wilk test was used to assess the distribution of the data for every data set. One-way ANOVA and independent *t*-tests were performed to determine statistical differences under different conditions. A Bonferroni adjustment for multiple comparisons was applied. If Mauchly’s test of sphericity was violated, Greenhouse–Geisser corrected values were used. Data are represented as the mean = 3, ± standard error of the mean (SEM). Significance was accepted at *p* ≤ 0.05.

## 3. Results

### 3.1. Phenolic Metabolites Improve Cell Viability by Increasing Akt Expression and Phosphorylation

**Figure 1:**
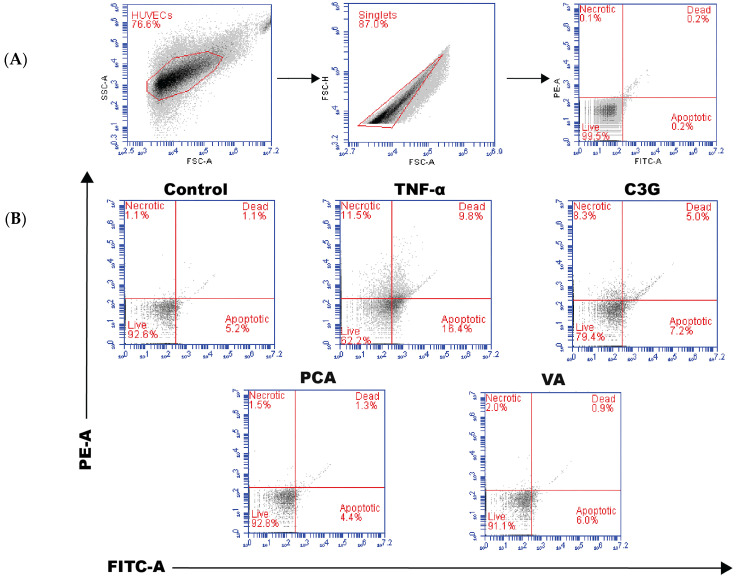

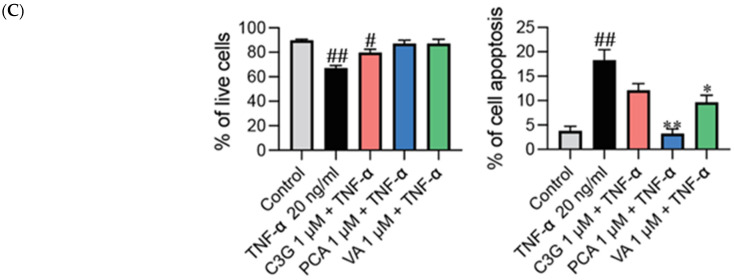
Phenolic metabolites prevent TNF-α induced apoptosis and maintain cell viability. HUVECs were pre-treated with C3G and phenolic metabolites for 24 h + stimulation with TNF-α for an additional 24 h to measure cell viability and apoptosis. (**A**) Flow cytometry analysis to determine cell viability and apoptosis in endothelial cells. (**B**) Representative data. (**C**) Representative graphs of quantitative analysis of % of live cells and cell apoptosis. All data expressed as mean ± SEM (n = 3). Significance value was set at # *p* < 0.05, ## *p* < 0.01 vs. control and * *p* < 0.05, ** *p* < 0.01 vs. TNF-α.

**Figure 2:**
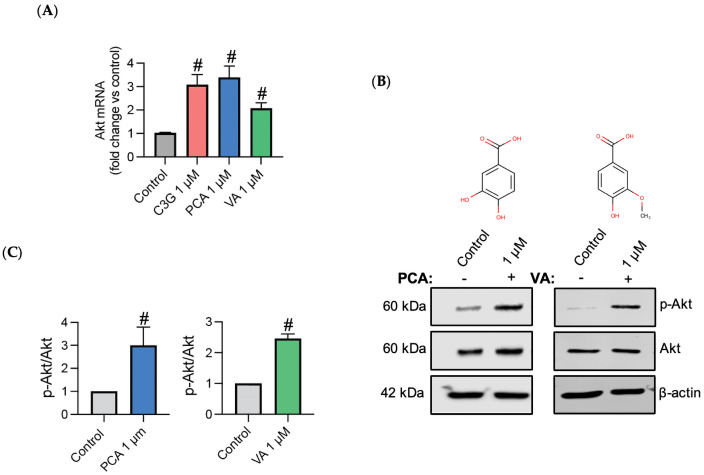
Phenolic metabolites upregulate Akt pathway. The expression and phosphorylation of Akt was measured in HUVECs stimulated with C3G and phenolic metabolites for 24 h. (**A**) Representative graphs for Akt mRNA expression levels. (**B**) Representative Western blot images for PCA and VA treatments. (**C**) Corresponding quantitative analysis graphs. All data expressed as mean = 3, ±SEM. Significance value was set at # *p* < 0.05 vs. control.

It has been well documented that TNF-α reduces endothelial cell viability through inducing apoptosis [25]. As expected, in our experiment, TNF-α induced endothelial cell apoptosis; however, pre-treating endothelial cells with C3G, PCA, and VA prevented TNF-α induced apoptosis and maintained cell viability (*p* < 0.05) (Figure 1). Previous reports have suggested that phenolic metabolites could stimulate cell survival signaling pathways [26,27]. To determine the effect of C3G and its phenolic metabolites on the Akt-eNOS pathway expression, qPCR analysis was carried out. In a non-inflammatory environment, Akt mRNA expression was upregulated following all treatments (*p* < 0.05) (Figure 2A). Given that C3G is known to activate the Akt-eNOS pathway at the protein level, we investigated whether phenolic metabolites could also stimulate this pathway. For further investigation, the effect of phenolic metabolites on Akt phosphorylation was measured at the protein level. Western blotting was carried out under the same conditions used to establish the gene expression (Figure 2B). Both PCA and VA were able to induce Akt phosphorylation at 1 μM (*p* < 0.05) (Figure 2C).

### 3.2. Phenolic Metabolites Prevent TNF-α-Induced eNOS Uncoupling but Do Not Modulate eNOS in Unstimulated Environment

**Figure 3:**
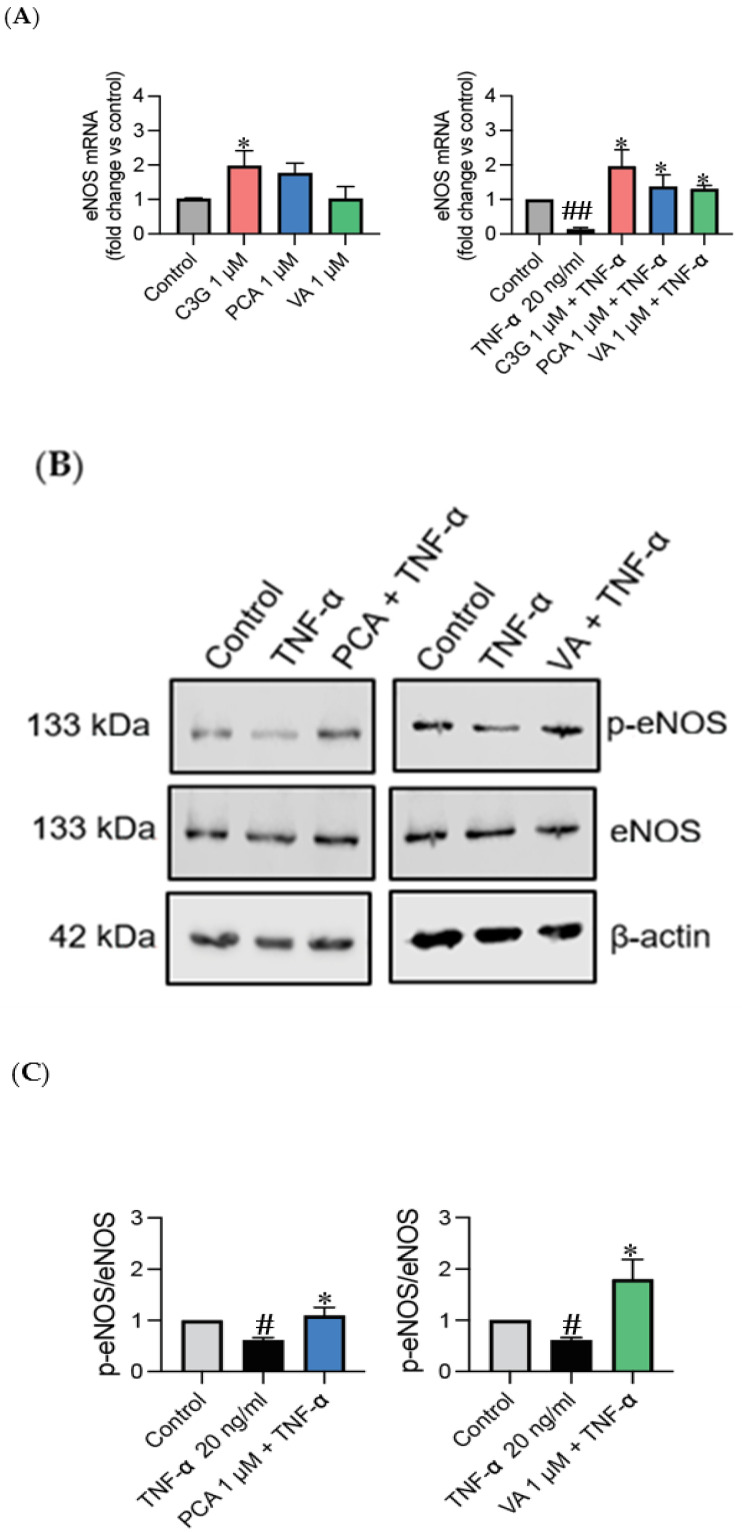
Phenolic metabolites upregulate eNOS pathway in response to TNF-α. The expression and phosphorylation of eNOS was measured in HUVECs pre-stimulated with C3G and phenolic metabolites for 24 h +/− TNF-α for an additional 24 h. (**A**) Representative graphs for eNOS mRNA. (**B**) Representative Western blot bands for eNOS. (**C**) Western blot representative graphs. All data expressed as mean = 3, ±SEM. Significance value was set at # *p* < 0.05, ## *p* < 0.01 vs. control and * *p* < 0.05 versus TNF-α.

Changes in eNOS mRNA levels can directly affect eNOS protein expression and subsequently impact NO bioavailability [28]. Therefore, by analyzing the eNOS mRNA levels, valuable insights can be gained regarding the transcriptional regulation of the eNOS gene expression in response to anthocyanin and phenolic metabolite treatment. TNF-α can reduce eNOS mRNA expression through transcriptional regulation, mRNA destabilization, and epigenetic modifications, thereby affecting the production of NO and potentially impacting endothelial function [29]. As expected, TNF-α was found to cause the downregulation of the mRNA expression of eNOS, as well as the downregulation of eNOS phosphorylation (*p* < 0.05) (Figure 3). Surprisingly, phenolic metabolites were able to cause the upregulation of eNOS mRNA expression in an inflammatory environment (Figure 3A), as well as prevent TNF-α induced eNOS dysfunction by inducing eNOS phosphorylation (*p* < 0.05) (Figure 3B,C). Despite this, PCA and VA failed to show an increase in eNOS expression at 1 µM without a TNF-α impairment (*p* > 0.05) (Figure 3A).

### 3.3. Phenolic Metabolites Mediate NO Bioavailability in Response to TNF-α

**Figure 4:**
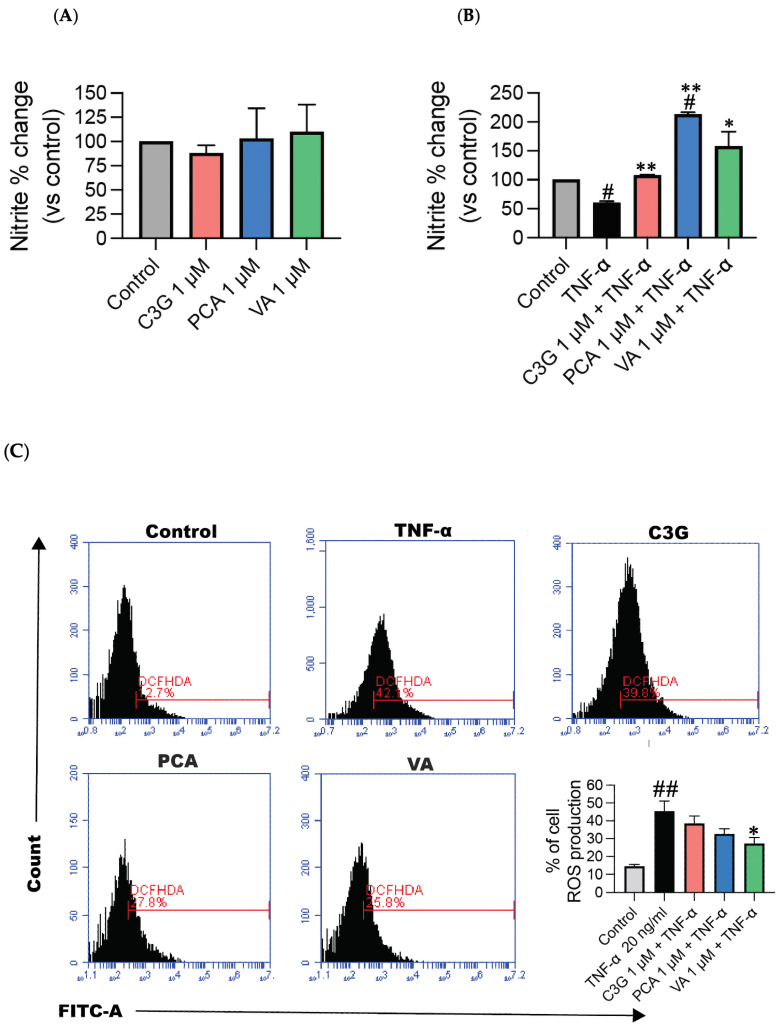
Phenolic metabolites regulate nitric oxide bioavailability. Nitrite levels and reactive oxygen species measured in supernatant of HUVECs pre-stimulated with C3G and phenolic metabolites for 24 h +/− TNF-α. (**A**) Representative graphs for NO—presence of TNF-α. (**B**) Representative graphs for NO + presence of TNF-α. (**C**) Representative images of DCFHDA assay. All data expressed as mean = 3, ± SEM. Significance value was set at # *p* < 0.05, ## *p* < 0.01 vs. control; * *p* < 0.01, ** *p* < 0.001 compared with TNF-α.

As expected, based on the eNOS mRNA measurements, the nitrite concentration was not increased following incubation with C3G, PCA, and VA (*p* > 0.05) (Figure 4A). However, stimulating HUVECs with TNF-α decreased NO production (Figure 4B), wherein pre-treating HUVECs with C3G and phenolic metabolites, prior to TNF-α stimulation, improved the NO production at 1 µM, following a similar trend to our eNOS experiments (*p* < 0.05) (Figure 4B). TNF-α is known to increase ROS, which can lead to oxidative stress. Oxidative stress impairs the bioavailability of NO by promoting the formation of peroxynitrite. To determine whether phenolic metabolites can mitigate TNF-α-induced ROS, we utilized a DCFHDA assay. TNF-α caused an increase in ROS production vs. the control (*p* < 0.05). Despite this, C3G did not lead to any reduction in ROS (*p* > 0.05), whereas both PCA and VA effectively reduced ROS production in response to TNF-α (Figure 4C).

## 4. Discussion

It is well established in the literature that anthocyanins, including C3G, can improve endothelial function by regulating the activity of the Akt-eNOS pathway, thus inducing NO production [30,31]. However, the low bioavailability of anthocyanins in vivo might suggest that the previously observed bioactivity may have been mediated by phenolic metabolites, which can reach higher concentrations and are in circulation for longer periods, rather than its parent anthocyanin structure [32,33]. The data presented in the current study demonstrate that the phenolic metabolites PCA and VA, at a physiologically relevant concentration of 1 μM, can improve NO bioavailability via the Akt-eNOS pathway in presence of TNF-α.

Recent in vivo studies have demonstrated that phenolic metabolites present in plasma correlate with improved markers of endothelial function [34,35,36]. A recent study revealed that the daily consumption of blueberry powder for 12 weeks improved endothelial function through reduced oxidative stress in postmenopausal women, and this was associated with metabolites detected in plasma [37]. Previous studies have indicated that an increase in the circulating concentrations of cyclic guanosine monophosphate (cGMP) shows no change detected in NO metabolites [38]. Nevertheless, accurately assessing the NO status is complex, and a single sample of blood may not reflect the NO tissue levels; thus, in the present study, it was crucial to investigate the mechanisms of action in vitro for further validation.

We investigated Akt for its involvement in cell proliferation, survival, and migration and as a downstream mediator of eNOS [39]. We found that PCA and VA induced the upregulation of Akt expression (Figure 2A), in addition to elevating its phosphorylation (Figure 2C), and this might have been linked to the observed improved cell viability and reduced TNF-α-induced apoptosis (Figure 1) [18]. In parallel to investigating the effect of Akt at the regulation level, the effect of phenolic metabolites on the expression of eNOS was established; similarly to other studies, it was found that no change or small changes were detected in a non-inflammatory environment (Figure 3A) [17]. Only studies that used a combination of metabolites demonstrated increased eNOS activation in human aortic endothelial cells in basal unstimulated conditions [40].

The novelty of our findings is that phenolic metabolites were able to prevent the impairment of eNOS in HUVECs at the regulation level (Figure 3B,C) by increasing eNOS phosphorylation and inducing NO production in response to TNF-α (Figure 4B). A recent study demonstrated that the incubation of db/db mouse aortas with a physiological concentration of PCA 100 nM ameliorated the endothelium-dependent relaxation impairment, as well as ROS overproduction mediated by diabetes [41]. Moreover, it was observed in human brain endothelial cells that PCA upregulated NO generation, with a major effect seen on the phosphorylation of eNOS, rather than at its protein levels, implying that this might also have happened in the current experiments [18]. Similarly, metabolites derived from Ginseng berry extract, which includes PCA, have also been reported to modulate NO production by inhibiting the intracellular accumulation of lipids and the overexpression of endothelin-1 (a vasoconstrictor), by enhancing Akt-eNOS phosphorylation [42]. Moreover, in a different study, treatment with VA attenuated the downregulation of eNOS and the upregulation of endothelin-1 expression in an inflammatory state [21].

TNF-α, as previously discussed, is a pro-inflammatory cytokine that has been previously shown to downregulate eNOS regulation, causing instability within the cytoplasm and a consequent reduction in NO production and impaired NO bioavailability [43,44]. However, we demonstrate that phenolic metabolites in the presence of TNF-α were able to induce NO production (Figure 4). Furthermore, as seen in the DCFHDA assay conducted in the current work, phenolic metabolites, but not the parent anthocyanin C3G, were able to suppress TNF-α-induced ROS production (Figure 4C), and this alteration could reduce the scavenging of NO and improve its bioavailability [45]. Similarly, in isolated human platelets, PCA, but not C3G, at physiologically relevant concentrations, was able to attenuate H_2_O_2_-induced apoptosis by downregulating ROS and apoptosis [46].

## 5. Conclusions

Overall, the current study demonstrates that the bioactivity of the phenolic metabolites PCA and VA may differ from that of their parent compound, C3G. Furthermore, the findings of the present work reveal that the induction of inflammation is a prerequisite for phenolic metabolites to promote protective properties in endothelial cells by activating the Akt-eNOS pathway. However, because the current study only assessed the independent effects of C3G, PCA, and VA, future studies should focus on a mixture of these and related metabolites’ responses to inflammation and/or oxidative stress.

## Data Availability

The raw data supporting the conclusions of this article will be made available by the authors on request.
